# Empirical Study on the Relationship Between Vacation Schedule and Seafarers’ Fatigue in Chinese Seafarer Population

**DOI:** 10.3389/fpsyg.2022.838811

**Published:** 2022-03-21

**Authors:** Ji An, Wenting Gao, Runze Liu, Ziqi Liu

**Affiliations:** ^1^Merchant Marine College, Shanghai Maritime University, Shanghai, China; ^2^Maritime College, Beibu Gulf University, Guangxi, China

**Keywords:** vacation, fatigue, stress, seafarer, psychology

## Abstract

**Background:**

Fatigue is an important factor for the safety of ships. In order to alleviate fatigue of the seafarers, the STCW Convention (International Convention on Standards of Training, Certification, and Watchkeeping for Seafarers) has made many regulations on the working time of seafarers. At present, if a crew member takes only one day off at home before returning to work on the ship, the working time on the ship must be re-calculated again. If the time spent at home is not sufficient to allow the crew to recover, the regulations of only stipulating the working time, not stipulating the home vacation time, cannot guarantee the crew’s fatigue been well controlled. The aim of present study is to explore the relationship between vacation schedule and fatigue of the seafarers.

**Methods:**

In present study, a simplified stress scale developed by the Ministry of Labor of Japan has been used as a measurement tool. The method of stratified sampling was adopted. Data collection mainly came from domestic ocean-going seafarers (*n* = 165). Analysis was conducted using the Cross (chi-square) analysis and hierarchical multiple regression analysis methods.

**Results:**

We found that there was no difference between crew members of different positions in terms of average vacation time and on-board service time (*p* > 0.05). The length of last vacation time and this service time for seafarers of different positions showed obvious differences (*p* < 0.01). The rank has a significant effect on the length of the last vacation (χ^2^ = 101.560, *p* = 0.000 < 0.01) and the length of this service time (χ^2^ = 75.624, *p* = 0.000 < 0.01). Also, the results showed that there was a significant negative correlation between the duration of vacation and overall fatigue (*t* = –7.160, *p* = 0.000 < 0.01), while there was a significant positive correlation between the length of service time on board and overall fatigue (*t* = 3.474, *p* = 0.001 < 0.01).

**Conclusion:**

The results indicated that a reasonable vacation schedule was crucial for the relief of the seafarers’ fatigue, and also played a positive role in the state of working on the ship again.

## Introduction

The shortage of seafarers, especially of young officers, is a continuing source of concern in the global shipping industry (Caesar et al., 2015). As a result, the seafarers on-board ships may work longer hours onboard, and in some cases, and receive less vacation periods (BIMCO/ISF, 2005). The physical and mental health and rights of seafarers need to be better protected (Nittari et al., 2019, 2020). Moreover, changes to port infrastructures and stricter international security would result in a reduction in shore leave. Especially because of the outbreak of COVID-19 in December 2019, it has prevented seafarers from disembarking to carry out crew changes, which resulted in seafarers serviced on board ship being extended for many months at sea, well beyond the established limits (Doumbia-Henry, 2020). Haka et al. (2011) found that the major reasons for leaving seafaring are the following: spending a long time away from home and family, problems posed by cultural differences, isolation or loneliness among officers. As a result, it appears to be a vicious cycle. A shortage of crew members has made shift changes difficult, leading to more working hours and less time off for crews (Ministry of Transport of the People’s Republic of China, 2022). This leads to less time for the crew to connect with family gatherings. Lacking of communication with family will lead to increased crew stress, work-family conflict, and decreased satisfaction (An et al., 2020). Crew satisfaction further affects turnover rates (Ulrich and Smallwood, 2007), leading to crew shortages. Practices such as these are both localized and global in nature and may impact on work efficiency and more importantly may create circumstances where maritime safety is impaired (Nguyen et al., 2014). So from the perspective of human resources, reducing vacation time and increasing service time on ship is not a good strategy to deal with crew loss.

Less vacation time and more service time on board means greater separation from family. Separation increases loneliness among seafarers and when coupled with fatigue and stress creates mental depression-a cause of suicide among seafarers (Iversen, 2011). Besides, separation with partners is stressful for both seafarers and their partners, leading to the loss of a critical psychogenic protective factor onboard (Oldenburg and Jensen, 2012). Some researchers acknowledge that separation also creates stress, fatigue, which result in high attrition among seafarers (Oldenburg et al., 2009; Haka et al., 2011). In shipping industry, fatigue is the main cause of stranding and collisions than other factors, at 53 and 38% respectively (Bloor and Thomas, 2000). Recovery from fatigue requires rest. To protect the rights and interests of ocean-going seafarers and prevent excessive stress and fatigue of seafarers, the shipping industry has made great efforts. Maritime Labor Convention 2006 (MLC2006) has specified the hours of work and rest for seafarers on board, stipulated the maximum service period on board shall not exceed 12 months. In fact, international conventions do not make it mandatory for seafarers about duration of vacation. The relevant rights and interests of seafarers are difficult to be guaranteed. Exceeded overtime hours worked by crew may be adjusted downwards by officers, and crew may therefore not receive pay for exceeded overtime hours. By under-reporting working hours, the shipping company may therefore stay within the limits of regulated working hours (McVeigh and MacLachlan, 2019). Similarly, an ITF report (2017) found that the employment of migrant fishers in Ireland is characterized by low pay, excessive hours and underpayment. The contracts identified by the ITF constituted a significant underestimation of the number of hours involved in fishing and extremely low pay. The effect of seafarers’ vacation time and service time cannot be positively guaranteed. From the above statement, it is particularly important to study the relationship between vacation and fatigue.

This paper mainly discusses the relationship between seafarer’s vacation and fatigue through investigation and research. This paper adopts the QSQ simplified scale (Kawakubo et al., 2017) developed by The Ministry of Labor of Japan as the basis for improvement in line with the professional characteristics of seafarers. The results of the survey can provide a better management of leave for shipping companies, so as to ensure the high quality of the crew working on the ship and protect the safety of ocean transportation. It can also provide reference for the further revision and improvement of the legal provisions on the protection of seafarers’ rights and interests.

## Literature

### Seafarer Fatigue

Crew fatigue has been recognized by the international community as an important factor threatening maritime safety (Dorrian et al., 2011; Akhtar and Utne, 2014). Fatigue represents a state of physical and/or mental weakness in a medical sense. It is also a symptom accompanying numerous diseases and one of the most frequent reasons for seeking medical attention (Kaltsas et al., 2010). At the same time, fatigue is associated with special working conditions. It is often divided into physical fatigue, which is the weakness and loss of endurance caused by prolonged physical activity, and mental fatigue, which is mainly due to mental stress and emotional fatigue, or the consequence of high workload such as working long hours. It has been shown that stress is one of the major causes of fatigue in individuals, and the higher the stress the higher the level of fatigue in individuals (Doerr et al., 2015). Stressful stimuli are responded to by multiple systems in the body (Southwick et al., 1999), and the coping responses of these mechanisms can lead to fatigue in the organism. Attention to the stressor continuously depletes the individual’s psychological resources and produces ego depletion (Baumeister et al., 2007). Therefore, this paper takes stress as an important consideration when studying fatigue. The International Maritime Organization (IMO) defines fatigue as “a decline in physical and/or mental abilities resulting from physical, mental, or emotional depletion, which may impair almost all physical abilities, including: strength; speed; reaction time; decision making; or the balance” (IMO, 2002). In a survey of seafarers, fatigue symptoms were found to be associated with a range of occupational and environmental risk factors at sea (sleep quality, hours of work, voyage time, job requirements, job stress, crossing time zones, etc.) (Jepsen et al., 2015). Therefore, fatigue seems to be very common in the shipping industry and may jeopardize vigilance at work (Dohrmann and Leppin, 2017). According to Härmä et al. (2008), 40.6% of the seafarers surveyed had fallen asleep on the job at least once in the past five years. Fatigue is also likely to be related with the safety of the crews, the ship and the environment (Allen et al., 2008). It was found in an interview of the crew’s collision experience, most crew members believe that fatigue is a potentially important factor for the high incidence of these accidents (Wellens et al., 2005). In the survey of more than 1,800 professional seafarers, 1/4 of them said that they would feel tired or sleepy at work, and nearly half of them believed that fatigue would reduce their awareness of collision (Wadsworth et al., 2006). Thus, to enhance health and well-being of seafarers and to avoid accidents and disasters, preventing fatigue is of great importance.

In order to reduce the risk of crew fatigue, modern merchant ships first adopt a large number of modern technologies to reduce human workload (Nilsson et al., 2009). Secondly, since the 1980s, the international community has made a lot of efforts. First of all, from the perspective of international conventions, many conventions have been formulated and implemented. For example, since the early 1990s, the IMO has adopted a number of resolutions on fatigue factors and guidelines on the mitigation and management of fatigue aimed at reducing the risk of fatigue by limiting the working hours and rest periods of seafarers. The International Labor Organization (ILO) Convention on “Working Hours of Seafarers and Manning of Ships” became mandatory in 2002. In June 2010, the Diplomatic Conference of the States Parties to the STCW Convention held in Manila, Philippines adopted the Manila Amendment to the STCW Convention, which came into force on January 1, 2012. The STCW Convention and the Maritime Labor Convention 2006 (MLC Convention) have detailed minimum requirements for seafarers’ work and rest standards. Reducing seafarers’ fatigue requires external and company regulation and control as well as individual preventive intervention and human resilience. Fatigue mitigating factors include alertness management strategies of which proper work-rest scheduling and adequate sleep hygiene are of primary importance (Caldwell and Caldwell, 2008).

### Vacation

Seafarers belong to a special class of occupation, where some evidence from neuroscience shows that the seafarers’ special occupational neuroplasticity (Wang et al., 2017, 2018; Wu et al., 2020; Shi et al., 2021; Yan et al., 2022). The working environment and tasks on board are very different from any onshore workplace (Oldenburg and Jensen, 2019). Seafarers must stay on ships for months and away from their families. As the shortage of crew worsens, this phenomenon is becoming more and more serious. However, the isolation, stress and work-family conflict caused by long periods away from home exacerbate the turnover rate. In addition, onboard workload, sleep, physical factors (vibration, noise), jet lag, etc., all contribute to crew fatigue and stress. Research in occupational health psychology has consistently shown that workplace stress has a negative impact on the health and well-being of individuals (Hjortskov et al., 2004; Belkic et al., 2014; Shi et al., 2015). It poses a serious threat to the navigation safety of ships and Marine environmental pollution. Among the strategies to solve the fatigue and stress of seafarers, many scholars have proposed to reduce working hours, improve seafarers’ working conditions, and provide free Internet for seafarers to contact with family and friends (Nguyen et al., 2014). In addition, some scholars, guided by work performance, suggested increasing the salary level of seafarers, providing good promotion channels, and providing professional training (Nguyen et al., 2014; Yuen et al., 2018). These measures are helpful to enhance seafarers’ job satisfaction and reduce seafarers’ psychological stress and fatigue to a certain extent.

However, there is a growing awareness of the great importance for recovery during non-working hours to protect workers from the adverse effects of work stress (Jessica et al., 2009; Kühnel and Sonnentag, 2011). As a longer period of uninterrupted time off, a holiday helps employees fully recover from work more than shorter breaks such as evenings or weekends (Eden, 2001; Etzion, 2003). This is not only useful for stress and burnout, but also both general fatigue and physical fatigue (Hooff et al., 2007; Yung et al., 2014). Earlier vacation studies have shown that vacations have a positive impact, with significant improvements in employees’ health and well-being during vacations (Westman and Etzion, 2001). A previous study showed that there was a negative relationship between working hours and happiness in China, Japan, and Taiwan (Yamashita et al., 2016). A reduction in overtime and restriction of excessive working hours could reduce work-related stress and prevent long-term illness (Westman and Etzion, 2001; Härmä, 2006). On the contrary, taking vacations could help to relieve job stress in people who work full-time, contributing to their overall life satisfaction (Chen et al., 2016). Vacations also had a great help to maintain people’s work ability, health and happiness levels (Jessica et al., 2012; Jessica and Geurts, 2014). Strandberg et al. (2017) showed that less vacation hours is a marker of higher mortality through analyzing of middle-aged men in long term. Kawakubo and Oguchi (2019) illustrated that recovery experiences during vacations promoted employee creativity and improved occupational well-being. Although it is widely believed that holidays are good for returning from work to the pre-holiday stage (Jessica et al., 2009), it is not clear whether the beneficial effects of holidays apply to all holidaymakers (Jessica et al., 2011). More and more crew members are taking less time off than ever before, a longer-term contract is needed ensure the crew numbers working on the ship. Especially in the case of the COVID-19, making the crew leave more difficult. According to a Statement on seafarers’ repatriation issued by the spokesperson for the Secretary-General in June 2020, hundreds of thousands of the world’s 2 million seafarers have been stranded at sea for months due to travel restrictions related to COVID-19. Unable to disembark, the maximum time at sea set by international conventions was ignored, leaving some sailors stranded at sea for up to 15 months. According to a report by the World Maritime University, seafarers are experiencing greater psychological distress than ever before and suicides are more frequent. The United Kingdom charity Seafarers’ Hospital Society has issued a message saying that suicide has become the number one source of deaths on board ships following the outbreak. The charity warned that the mental health of seafarers is of concern with many reported suicides and suicide attempts on board ships stranded at sea or in port. This made us realize the importance of looking at crew leave not only during outbreaks, but also during normal times. This is also the purpose of this study on the relationship between vacation and fatigue.

## Materials and Methods

### Participants

In present study, a stratified random sample of 165 ocean-going seafarers was used as case study. The participants is this study are all from China. The questionnaire had the relevant ethical approval, was distributed via the internet, and ensured that all crew members were informed that the study was being conducted. Due to the epidemic, it is difficult to investigate the foreign crew members when they are under port state surveillance, and there is a lack of other contact information with the foreign crew members. Participants are working in different ship management companies. In order to make the data more real and effective, data of sample came from a large state-owned shipping companies and several small intermediary companies which are with a different team of the flag state, such as Chinese, Hong Kong and Panama, operates a different route, China’s coastal, southeast Asia routes and ocean routes, such as Europe, America line. The crew is from large bulk carriers (60,000-180,000 tons) and container ships (3,000–12,000 slots). This sample was stratified by rank and composed of 32 cadets (19.4%), 44 support class crews (26.7%), 80 operation class crews (48.5%) and 9 management class crews (5.4%). The age of the seafarers ranged from 18 to 64 years old (M = 36.80, *SD* = 9.75), 30–39 have the largest number of people (67 subjects, 40.6%). From the perspective of marital status, it can be seen that the numbers of unmarried (29.1%) and married (70.9%) samples. The collected questionnaires cover various regions. Shanghai has the largest number (38.2%), followed by Guangzhou (8.48%) (see Table 1).

**TABLE 1:** Cross-tabulation of demographic variables.

Demographics	Category	Rank (%)				Total	χ^2^	*p*
		Cadet	Support	Operation	Management			
Company location	Guangzhou	3(9.38)	4(9.09)	7(8.75)	1(11.11)	14(8.48)	25.539	0.043*
	Shanghai	20(62.50)	11(25.00)	26(32.50)	6(66.67)	63(38.18)		
	Other regions	9(28.12)	29(65.91)	47(58.75)	2(22.22)	88(53.33)		
Total		32	44	80	9	165		
Age	18–29	11(34.38)	20(45.45)	10(12.50)	0(0.00)	41(24.85)	78.134	0.000**
	30–39	21(65.63)	15(34.09)	29(36.25)	2(22.22)	67(40.60)		
	40–59	0(0.00)	9(20.46)	40(50.00)	5(55.56)	54(32.73)		
	59+	0(0.00)	0(0.00)	1(1.25)	2(22.22)	3(1.82)		
Total		32	44	80	9	165		
Marital status	Unmarried	12(37.50)	15(34.09)	1(1.25)	0(0.00)	48(29.09)	112.286	0.000**
	Married	20(62.50)	29(65.91)	79(98.75)	9(100.00)	117(70.91)		
Total		32	44	80	9	165		

* p<0.05, **p<0.01.

### Measuring Tools

A simple occupational stress scale has been developed by the Stress Measurement Team of The Japanese Labor Department (Department of Labor of Japan, 2000). This scale was chosen in consideration of culture and other factors, since the survey was conducted on Chinese sailors. About 2500 workers were taken as participants to study their credibility. The results showed that their credibility and relevant reliability was relatively high. The author has managed people from different cultural backgrounds as a chief engineer, and based on his sailing experience, people from different cultures have very different tolerance for stress and fatigue (Masuda and Nisbett, 2001, 2006; Jiang et al., 2006; Boduroglu et al., 2009). Relatively speaking, people in the East Asian culture have more in common on this issue. Therefore, this scale was chosen for this paper (Gu, 2009). The corresponding factor structure was also recognized. The scale can be divided into three dimensions to show the stress and fatigue of the subjects. These are stress response (anxiety), physical fatigue symptoms and communicative support.

In order to reduce the length of the original version, Kawakubo et al. (2017) ruled out stress projects related to work and family, friends and colleague support. Then, nine items related to fatigue and anxiety were used. Participants rated their stress and fatigue status on a scale of 1 = not at all, with 4 = almost always. Higher scores mean more fatigue. Items contain “I feel tired,” “Everything is a burden,” “I feel insecure,” and so on. The specific contents of the scale are as follows:

#### Simplified Version of the Occupational Stress Questionnaire

Please circle the point on the scale that applies to your state today.

1 = Not at all  2 3 4 = Most or all the time

(1) I feel tired.
(2) I feel exhausted.
(3) I feel lazy.
(4) I feel strained.
(5) I feel uneasy.
(6) I feel anxious.
(7) I feel depressed.
(8) Everything is burdensome.
(9) I’m in a bad mood.

### Questionnaire Design

The questionnaire contains 17 questions which were mainly divided into three parts. The first four questions were mainly collected as basic information, related to the seafarers’ position, age, marital status, company location (Q1–Q4). In addition to demographics, questions such as “the duration of the last vacation, duration of this service on ship, average time spent on the ship and average vacation time at home” were also designed (Q5–Q8). Vacation refers to the period during which a crew member is on leave at home. The first two items were used as vacation evaluation items, while the second two items could be used for basic statistics of Chinese seafarers’ vacation. The last 9 items are fatigue items of seafarers based on the simplified version of occupational stress scale (Q9–Q17) (see Appendix Table A).

### Data Collection and Analysis

The data used in present study were collected from several ship management companies in different regions of China. Participation was anonymous and voluntary written consent was obtained from all participants. The study was approved by the Ethics Committee of the Shanghai Maritime University. Questionnaires were distributed via email to all 10 of the Chinese companies’ vessels that were at sea. In order to get more precise answers and a higher response rate, the questionnaire was translated into Mandarin prior to distribution to the Chinese seafarers. The translated and reverse translated versions were then compared in order to verify discrepancies and make corrections (Almeida and Freire, 2007). Questionnaires were distributed to seafarers at sea in March and April 2020. Off the total 203 seafarers on the 10 ships, 195 exclusively male seafarers took part in this survey (a participation rate of 96%). Since each department (Deck department, Engine department, and Service department) may differ in its response to fatigue, our data include any of them. In total, after eliminating invalid questionnaires, the remaining 165 questionnaires were valid (84.6%). The invalid questionnaires were mainly due to incomplete data filling or inconsistent answers to questions about fatigue.

Exploratory data analysis showed that, for the majority of the variables, the assumptions for using parametric tests were satisfied (Simães et al., 2019). Construct validity was assessed by exploratory principal components factor analysis. The internal consistency reliability was calculated by Cronbach’s Alpha Coefficients (Maroco, 2014). Statistical tests were carried out using SPSS Statistics.

## Results

### Chi-Square Analyses

Table 1 shows that using the chi-square test (cross analysis) to study the three different relationships between rank to the company location, age, marital status. It can be seen from the table above: samples of different ranks for the company location (χ^2^ = 25.5, *p* = 0.000), age (χ^2^ = 78.1, *p* = 0.000), and marital status (χ^2^ = 112.3, *p* = 0.000) presents a significant (*p* < 0.01). This means that the location, age and marriage of different rank samples are different from each other which has good statistical significance.

### Testing of Validity and Reliability

The conceptual structure of the scales was achieved by replicating the proceedings described in the original study. A principal components factor analysis of items has been made, using orthogonal rotation and eigenvalue ≥ 1. The Kaiser–Meyer–Olkin index (KMO ≥ 0.6) and the Bartlett’s test (*p* < 0.05) indicated the sample’s adequacy for this procedure (KMO = 0.866; χ^2^ = 878.654, df = 36, *p* = 0.001). These indicated that correlation matrix was not an identity one and factor analysis was able to be carried out (Kaiser, 1974). The exploratory factor analysis demonstrated that the items of the instrument are organized by three dimensions. Together, the three factors explain 79.7% of the total variance found in this study. After rotation, factor I was composed of the first two items (Q9, Q10) and justifies 11.3% of the overall founded variance. Factor II was comprised of the middle three items (Q11–Q13), contributing with 13.5% of the total explained variance. Factor III was composed of the last four items (Q14–Q17) with 54.9% of the total explained variance. All the items revealed factorial weights above 0.50 and were grouped in only one factor. Results for the construct validity of the new scale are in Table 2.

**TABLE 2:** Construct validity and reliability results for the new scale (*N* = 165).

Items	Factor Load			Item-Total Correlation	α if Item Deleted
	I	II	III		
Q9 I feel tired on board	0.923			0.439	0.899
Q10 I feel exhausted at the end of day	0.773			0.642	0.884
Q11 I feel that I am not actively engaged in my work		0.866		0.676	0.881
Q12 I’m afraid I’m not up to the job		0.809		0.693	0.880
Q13 Everything feels like a burden		0.826		0.689	0.880
Q14 Working and living on a ship is very stressful			0.895	0.669	0.882
Q15 I often feel uneasy during the ship			0.776	0.706	0.879
Q16 I feel anxiety during life on board			0.814	0.677	0.881
Q17 I feel depressed during the boat trip			0.774	0.720	0.878
Eigenvalues			1.13	1.35	5.49
Total Explained Variance					79.7%

*Orthogonal rotation with Kaiser standardization, Principal component analysis.*

The reliability for the instrument showed that the Cronbach α value of the new scale was 0.89. This means a good internal consistency reliability of the scales. Table 2 also showed the item-total correlations and the Cronbach α value if item removed. The correlation for each item was between 0.878 and 0.899, indicating that all items contributed significantly to the overall measurement.

### Preliminary Data Analysis

The chi-square test (cross analysis) was used to study the differences among the four items, including Q5: the duration of the last vacation, Q6: the duration of this service, Q7: how long you work on the ship for each vacation, and Q8: how long you take each vacation. From Table 3, the samples of different ranks did not show significance (*p* > 0.05) in Q7 and Q8, implying that there was no difference between crew members of different positions in terms of average vacation time and length of service on board. In addition, for Q5 and Q6 were significant (*p* < 0.01) in the sample of positions. In recent years, with the loss of seafarers, especially young senior seafarers, the last leave time and the current service time of seafarers in different positions showed significant differences.

**TABLE 3:** Cross (chi-square) analysis results of vacation situation of each position.

Items(length of time)		Rank(%)				Total	χ^2^	*p*
		Cadet	Support	Operation	Management			
Q5 Last vacation	<1 month	0(0.00)	0(0.00)	2(2.50)	0(0.00)	2(1.21)	101.560	0.000**
	1–2 months	0(0.00)	25(56.82)	52(65.00)	7(77.78)	84(50.91)		
	2–3 months	12(37.50)	18(40.91)	26(32.50)	2(22.22)	58(35.15)		
	>3 months	20(62.50)	1(2.27)	0(0.00)	0(0.00)	21(12.73)		
Total		32	44	80	9	165		
Q6 This service	1–3 months	20(62.50)	5(11.36)	1(1.25)	0(0.00)	26(15.76)	75.624	0.000**
	3–6 months	12(37.50)	17(38.64)	33(41.25)	3(33.33)	65(39.39)		
	6–9 months	0(0.00)	22(50.00)	46(57.50)	6(66.67)	74(44.85)		
Total		32	44	80	9	165		
Q7 AVE service	6–7 months	9(28.13)	8(18.18)	10(12.50)	5(55.56)	32(19.39)	16.827	0.051
	7–8 months	13(40.63)	14(31.82)	25(31.25)	1(11.11)	53(32.12)		
	8–9 months	5(15.63)	12(27.27)	30(37.50)	1(11.11)	48(29.09)		
	>9 months	5(15.63)	10(22.73)	15(18.75)	2(22.22)	32(19.39)		
Total		32	44	80	9	165		
Q8 AVE vacation	<1 month	0(0.00)	0(0.00)	1(1.25)	0(0.00)	1(0.61)	13.370	0.147
	1–2 months	9(28.13)	9(20.45)	7(8.75)	0(0.00)	25(15.15)		
	2–3 months	9(28.13)	13(29.55)	17(21.25)	3(33.33)	42(25.45)		
	>3 months	14(43.75)	22(50.00)	55(68.75)	6(66.67)	97(58.79)		
Total		32	44	80	9	165		

*p<0.05, **p<0.01.

The rank had a significant effect on the length of the last vacation (χ^2^ = 101.560, *p* = 0.000 < 0.01). Through the percentage comparison, it could be seen that the proportion of management grade and operation grade choosing one month was 77.78%, 65.00% respectively, which was greatly higher than the average level of 50.91%. The proportion of supporting grade choosing 2 months was 40.91%, which was significantly higher than the average level of 35.15%. The proportion of cadets who choose three months or above was 62.50%, which was dramatically higher than the average level of 12.73%. As it could be seen from the above, officers took less time off at home than ordinary crew.

The rank also had a great effect on the length of this service time (χ^2^ = 75.624, *p* = 0.000 < 0.01). According to the percentage comparison, 62.50% of cadets chose 1–3 months, which was significantly higher than the average level of 15.76%. The proportion of management grade and operation grade choosing 6–9 months was 66.67 and 57.50% respectively, which was significantly higher than the average level of 44.85%. It showed that the officers generally work long hours on the ship and are not easy to change shifts.

The single-sample *t*-test was used to analyse the overall fatigue of the crew, and the input fatigue threshold was 2.5 (Li and Gong, 2021). Fatigue values above 2.5 indicated that the crew was in a state of fatigue. It was found that nine items for measuring fatigue were significantly greater than 2.5, and all of them showed significant significance (*p* < 0.01). The average fatigue value of the nine items in the questionnaire was between 2.71 and 2.95 (*M* = 2.83, *SD* = 0.991). So the crew was indeed in a state of fatigue. There was no denying that the timing of our investigation coincided with the outbreak, which may have exacerbated the fatigue scores to some extent (see Table 4).

**TABLE 4:** The single sample *t*-test analysis results of fatigue items.

Items	*N*	Min	Max	*M*	*SD*	*t*	*p*
Q9 I feel tired on board	165	1.000	4.000	2.945	0.958	5.973	0.000**
Q10 I feel exhausted at the end of day	165	1.000	4.000	2.879	0.968	5.028	0.000**
Q11 I feel that I am not actively engaged in my work	165	1.000	4.000	2.879	1.125	4.325	0.000**
Q12 I’m afraid I’m not up to the job	165	1.000	4.000	2.733	0.995	3.013	0.003**
Q13 Everything feels like a burden	165	1.000	4.000	2.709	0.937	2.866	0.005**
Q14 Working and living on a ship is very stressful	165	1.000	4.000	2.909	1.075	4.887	0.000**
Q15 I often feel uneasy during the ship	165	1.000	4.000	2.788	1.017	3.637	0.000**
Q16 I feel anxiety during life on board	165	1.000	4.000	2.885	0.920	5.373	0.000**
Q17 I feel depressed during the boat trip	165	1.000	4.000	2.752	0.927	3.487	0.001**

*p<0.05, **p<0.01.

### Hierarchical Regression Analysis

Hierarchical regression was used to study the model changes brought about by the increase of independent variable, which was usually used to test the stability of the model and study the mediating effect or regulating effect (Mu and Tian, 2020). It could be seen from the table that this hierarchical regression analysis involved two models. In Model 1, the independent variables were rank, company location, age and marriage. In Model 2, based on Model 1, the length of last vacation and the length of this service time were added. The dependent variable of the model was: overall fatigue.

From Table 5, it could be seen that, the rank, company location, age and marriage were taken as independent variables and the overall fatigue was taken as dependent variable for linear regression analysis. The R-square value of the model was 0.418, which meant that the rank, company location, age and marriage can explain 41.8% of the change in the overall fatigue. The *F*-test on the model showed that the model passes the *F*-test (*F* = 28.733, *p* < 0.05), that was, at least one of the rank, company location, age, and marriage will have an impact on the overall fatigue, and the model formula was: overall fatigue = 1.412 + 0.547* position + 0.116* company location + 0.013* age –0.232* marriage. The regression coefficient of rank was 0.547 and showed a significant value (*t* = 6.329, *p* = 0.000 < 0.01), indicating that rank had a significant positive effect on overall fatigue. The regression coefficient value of the company location was 0.116, and it was significant (*t* = 4.602, *p* = 0.000 < 0.01), which meant that the company location had a significant positive influence on the overall fatigue. This may be related to the company’s human resource policies, such as vacation arrangements, promotions, compensation, and training. The regression coefficient of age was 0.013, which showed no significant difference (*t* = 0.130, *p* = 0.897 > 0.05), The regression coefficient of marriage was –0.232, and there was no significant correlation (*t* = –1.508, *p* = 0.133 > 0.05).

**TABLE 5:** The results of hierarchical multiple regression analysis (*n* = 165).

	Model 1				Model 2			
	β	*SE*	*t*	*p*	β		*t*	*p*
Constant	1.412**	0.225	6.287	0.000	3.478**	0.408	8.519	0.000
Rank	0.547**	0.086	6.329	0.000	0.077	0.061	1.255	0.211
Location	0.116**	0.025	4.602	0.000	0.029	0.017	1.742	0.083
Age	0.013	0.099	0.130	0.897	0.046	0.061	0.747	0.456
Marriage	–0.232	0.154	–1.508	0.133	–0.098	0.097	–1.017	0.311
Duration of last vacation					–0.571**	0.080	–7.160	0.000
Duration of this service					0.267**	0.077	3.474	0.001
*R* ^2^	0.418				0.779			
Adjusted *R* ^2^	0.403				0.771			
*F*	*F* (4,160) = 28.733, *p* = 0.000				*F*(6,158) = 92.821, *p* = 0.000			
△*R*^2^	0.418				0.361			
△*F*	*F*(4,160) = 28.733, *p* = 0.000				*F*(2,158) = 129.029, *p* = 0.000			

*Dependent variable: overall fatigue.*

* p<0.05, **p<0.01.

For Model 2, after the length of last vacation and service this time was added on the basis of Model 1. The change of *F*-value showed significant (*p* < 0.05), indicating that the length of last vacation and service this time had explanatory significance for the model. In addition, the R-square value increased from 0.418 to 0.779, indicating that the duration of last vacation and the duration of this service could account for 36.1% of the overall fatigue. Specifically, the regression coefficient of the length of last vacation was –0.571, and it was significant (*t* = –7.160, *p* = 0.000 < 0.01), which meant that the length of last vacation had a significant negative influence on the overall fatigue. The regression coefficient of the service length was 0.267, and it was significant (*t* = 3.474, *p* = 0.001 < 0.01), which meant that the service length would have a significant positive impact on the overall fatigue.

## Discussion

Through our study on fatigue, it was found that the crew was indeed in a serious state of fatigue. In the process of vacation statistics, most of the crews were found to have insufficient vacation and overworked on the ship, especially the senior crew. The higher the rank was, the greater the degree of fatigue was. Salyga and Kušleikaite (2011) showed that those working in the management sector were more likely to experience fatigue compared to others. It was undeniable that the position was in direct proportion to the responsibility, but it also slightly verified what has been mentioned above: the influence from insufficient leave of officers and serious overdue work on the ship. In addition, company location was positively correlated with fatigue, suggesting that the company’s HR policy might be a possible contributing factor to fatigue. Taking vacation time and working time into account in the situation, we found that vacation time was significantly negatively correlated with fatigue, while working time was significantly positively correlated with fatigue. Moreover, statistically speaking, increasing vacation time was more important than reducing working time on ship to reduce fatigue (*t* = –7.160, *p* = 0.000 for last vacation time, *t* = 3.474, *p* = 0.001 for service time). This result made us know the importance of reasonable vacation arrangement. It could not only strengthen the communication with family members, reduce the degree of separation, and help companies to retain the crew, but also become an effective strategy to alleviate crew fatigue. This point played a vital role in crew’s own life and health safety, and even the safety of the ship, which would cause sufficient attention from the seafarers’ companies, competent authorities and international regulations.

As with most research, the results of the present research must take into account certain limitations. Firstly, few previous studies had investigated effects of vacations on fatigue in the seafaring. Some of the existing studies illustrate the significance of vacation for seafarers from the perspective of crew retention (Nguyen et al., 2014; Caesar et al., 2015, 2021). Meanwhile, the study examining the determinant of fatigue had placed more emphasis on the psychological stress of separation from family on seafarers (Jepsen et al., 2015; Dohrmann and Leppin, 2017). Both seem to miss the effect of vacation time on crew fatigue, so the relevant studies foundation of our survey was insufficient. Secondly, the research method in this study belonged to subjective measurement, which was insufficient compared with objective measurement. Thirdly, this study was based on vacation time and did not take into account other events experienced by the crew while on vacation. Different individuals may experience fatigue differently due to various factors. Fourthly, the relatively small sample also limited us to control the impact of shipping routes on fatigue. Future research needs to take this into account. It is difficult to extrapolate from just one study to the entire maritime industry, which constitutes a wide diversity of States, employers, flags, ship types, contract types, recruitment and remuneration practices, distances and routes traveled, policies, practices, routines, multinational nature of the organization, and public profile of the organization – which all establish a particular working and living environment on-board that is not necessarily shared across shipping organizations or groups of seafarers. Finally, due to the outbreak, many crew members were forced to extend their service hours on board, which affected our findings to a certain extent.

## Conclusion

Overall, the fatigue of seafarers is a complex structure that may be manifested by various occupational factors. In this paper, findings illustrated that relatively more vacation time and less work time are important to alleviate seafarers’ fatigue. On the one hand, these results can help shipping companies develop better leave management systems and ensure high-quality work for seafarers on board. On the other hand, the research results have important guiding significance for shipping companies to strengthen the management system of leave and the protection of seafarers’ rights and interests. Factors affecting the well-being and performance of maritime workers exist at various work levels, including task, individual, team, organization and industry (Maclachlan et al., 2013). While the Maritime Labor Convention aspires to standards of good work practice, the broader shipping industry focuses on the “rationalization” of work practices, which is often at odds with, and may even run counter to, the spirit of the Maritime Commission. In order to make companies competitive, they may be forced to rationalize in ways that harm seafarers’ well-being, performance and safety. It is therefore crucial to establish incentives at the industrial level that can support the well-being of seafarers.

## Data Availability Statement

The raw data supporting the conclusions of this article will be made available by the authors, without undue reservation.

## Author Contributions

JA and WG conceived and designed the study and collected and analyzed the data. WG, RL, and ZL wrote the first draft. All authors contributed to later drafts and approved the final manuscript.

## Conflict of Interest

The authors declare that the research was conducted in the absence of any commercial or financial relationships that could be construed as a potential conflict of interest.

## Publisher’s Note

All claims expressed in this article are solely those of the authors and do not necessarily represent those of their affiliated organizations, or those of the publisher, the editors and the reviewers. Any product that may be evaluated in this article, or claim that may be made by its manufacturer, is not guaranteed or endorsed by the publisher.
