# Supplementary material for: Empirical Study on the Relationship Between Vacation Schedule and Seafarers’ Fatigue in Chinese Seafarer Population
Source: Front Psychol. 2022 Mar 21;13:838811. doi: 10.3389/fpsyg.2022.838811 (PMC8977519; doi:10.3389/fpsyg.2022.838811)

**Appendix**

**Table A Ocean-going seafarers’ vacation and fatigue scale**

| Dear crew friends, the purpose of this questionnaire is to conduct academic research, mainly to explore the relationship between ocean-going seafarers’ fatigue and vacation. The results can provide references for the seafarers’ vacation policy and strive for greater benefits for the seafarers. Thank you very much for your active cooperation. I wish you a smooth work and a happy life.  Information about the ship:   - - - 1. Type and size of your vessel _______________.       2. The route of the ship is ________________.       3. The flag state of the ship is ______________. |
| --- |
| The choices for questions 9 to 17 represent as follows: 1. none; 2 .sometimes; 3.in general; 4.all the time.   1. Your position on board [multiple choice]   ○ Cadet ○ Support ○ Operation ○ Management   1. Where is your company [multiple choice]   ○ Shanghai ○ Guangzhou ○ Other regions  3. How old are you [multiple choice]  ○ 18-29years old ○ 30-39years old ○ 40-59years old ○ > 59years old  4. Are you married or not [multiple choice]  ○ Married ○ Unmarried  5. How long was your last vacation [multiple choice]  ○ ＜1 month ○ 1-2 month ○ 2-3 months ○ >3 months  6. How long have you been working on this ship during this service period [multiple choice]  ○ 1-3 months ○ 3-6 months ○ 6-9 months  7. About how long you work on a ship every time [multiple choice]  ○ 6-7 month ○ 7-8 month ○ 8-9 months ○ >9 months  8. How long is your vacation approximately each time [multiple choice]  ○ ＜1 month ○ 1-2 month ○ 2-3 months ○ >3 months  9. I feel tired on board  ○ 1 ○ 2 ○ 3 ○ 4  10. I feel exhausted at the end of day  ○ 1 ○ 2 ○ 3 ○ 4  11. I feel that I am not actively engaged in my work  ○ 1 ○ 2 ○ 3 ○ 4  12. I'm afraid I'm not up to the job  ○ 1 ○ 2 ○ 3 ○ 4  13. Everything feels like a burden  ○ 1 ○ 2 ○ 3 ○ 4  14. Working and living on a ship is very stressful  ○ 1 ○ 2 ○ 3 ○ 4  15. I often feel uneasy during the ship  ○ 1 ○ 2 ○ 3 ○ 4  16. I feel anxiety during life on board  ○ 1 ○ 2 ○ 3 ○ 4  17. I feel depressed during the boat trip  ○ 1 ○ 2 ○ 3 ○ 4 |

**Appendix Certificate of Ethics Review**


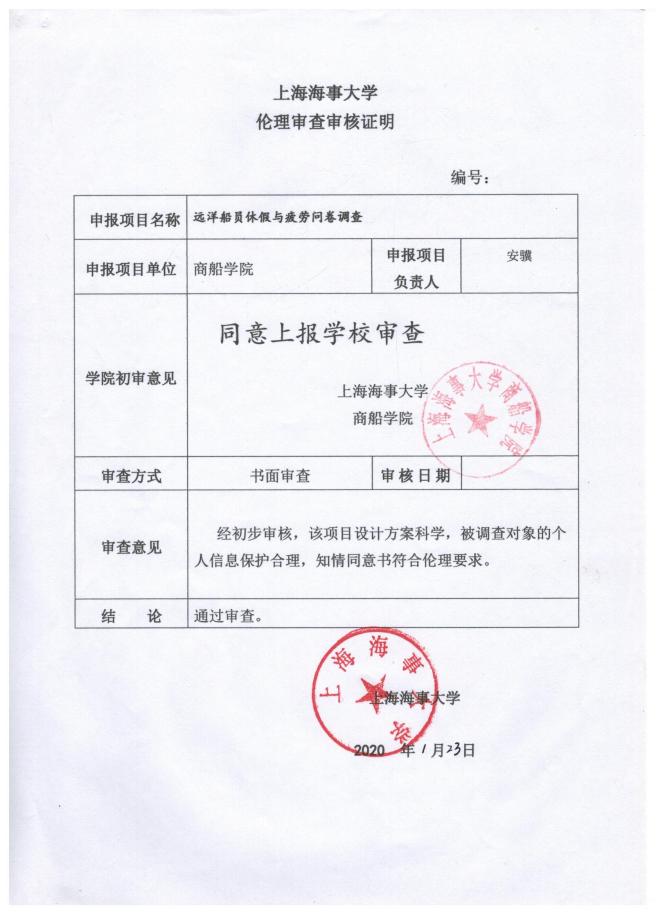

Supplement: Supplementary file 1 [file Data_Sheet_1.docx]
